# Evaluation of Degenerative Lumbar Scoliosis After Short Segment Decompression and Fusion

**DOI:** 10.1097/MD.0000000000001824

**Published:** 2015-10-30

**Authors:** Naiguo Wang, Dachuan Wang, Feng Wang, Bingyi Tan, Zenong Yuan

**Affiliations:** From the Department of Spinal Surgery, Shandong Provincial Hospital affiliated to Shandong University, Jinan, Shandong, China.

## Abstract

The objective of this study was to investigate short segment decompression of degenerative lumbar scoliosis (DLS) and the efficiency of fusion treatment.

After DLS surgery, the patients were retrospectively reviewed using the VAS (visual analog scale) and ODI (Oswestry Disability Index) to assess clinical outcomes. All patients underwent posterior lumbar decompressive laminectomy, pedicle screw internal fixation, and posterolateral bone graft fusion surgery. Radiographic measurements included the scoliotic Cobb angle, the fused Cobb angle, the anterior intervertebral angle (AIA), the sagittal intervertebral angle (SIA), and lumbar lordosis angle. The relationships between these parameters were examined by bivariate Pearson analysis and linear regression analysis.

Preoperatively, the Cobb angle at the scoliotic segment was 15.4°, which decreased to 10.2° immediately following surgery (*P* < 0.05). The AIA significantly increased by the last follow-up (4.4 ± 3.4) compared with pre- and postoperative values (2.5 ± 2.8 and 2.2 ± 2.4, respectively; *P* < 0.05). However, the scoliotic Cobb angle and the AIA did not correlate with the VAS or ODI scores. At the final follow-up, no patients had pseudoarthrosis or internal instrumentation-related complications.

Short fusion surgical treatment results in limited DLS correction, with correction loss over time. The AIA between the upper adjacent segment and proximal fused vertebra continues to increase postoperatively, which does not exacerbate clinical symptoms, as reflected by the low reoperation rates for repairing degeneration at adjacent levels.

## INTRODUCTION

Degenerative lumbar scoliosis is a three-dimensional spinal deformity in skeletally mature adults.^1,2^ Epidemiologically, degenerative lumbar scoliosis primarily involves elderly patients in the sixth decade of life, especially women. Degenerative scoliosis is usually caused by asymmetric degeneration of the intervertebral complexes, including the intervertebral disc and facet joint. These changes induce asymmetric and progressive lateral listhesis or rotation of the vertebra and ultimately lead to scoliosis, loss of lumbar lordosis, lateral or anterior vertebral translation, and lateral rotational subluxation.^1–3^

Degenerative lumbar scoliosis often occurs concomitantly with lumbar spinal stenosis; patients often present with low back pain (LBP), with or without neural injury symptoms, such as radiating radicular pain or myasthenic intermittent claudication.^4–6^ These symptoms are similar to lumbar spinal stenosis but may not be relieved with simple postural changes, such as anteflexion or sitting, and often require bed rest and surgical treatment.^4–6^

When conservative treatment fails in treating symptomatic degenerative lumbar scoliosis, surgery is often considered.^7^ The surgical treatment strategy includes decompression alone and decompression combined with spinal fusion (with or without internal fixation).^8,9^ However, there is no universal agreement about which levels to select for fusion. Patients with degenerative lumbar scoliosis who choose to undergo surgical treatment mainly do so because of symptoms that are related to lumbar spinal stenosis; stenosis generally involves a shorter segment of the spine than scoliosis. Although longer-segment spinal fusion is more stable, this approach results in a decreased number of mobile segments, decreased spinal range of motion, and increased costs. As patients with degenerative lumbar scoliosis represent a special population of elderly patients with multiple medical comorbidities, extended fusion procedures can unnecessarily increase the risk of complications, such as bleeding and complications related to internal fixation.^10–12^ When a patient's general condition is taken into account, long-segment fusion may not necessarily be the best approach. Often, a patient's symptoms may be alleviated by short segment decompression and fusion surgery that is limited to the upper and lower vertebral boundaries of the scoliotic region.^13,14^

To the best of our knowledge, there are several researches analyzing the efficacy of different fusion approaches for degenerative lumbar scoliosis,^15–18^ but there are no standards of care or published reports analyzing the efficacy of short-segment decompression and fusion of degenerative lumbar scoliosis. Therefore, the aim of this study was to assess the feasibility and efficacy of short-segment decompression and fusion of degenerative lumbar scoliosis.

## MATERIALS AND METHODS

Thirty-one patients with degenerative lumbar scoliosis, who were treated by the same surgeon between June 2004 and November 2011, were reviewed. Of the 31 patients, 6 were men and 25 women. The research was approved by the ethical committee of Shandong Provincial Hospital affiliated to Shandong University. Written consents were obtained from all patients. The mean age of the patients was 60.7 years (range 45–80) (Table 1). Scoliosis involved 3 to 7 vertebrae, with an average of 5.0 vertebrae. The highest upper end vertebra (UEV) was at T_11_, and the lowest at L_2_. The highest lower end vertebra (LEV) was L_4_, and the lowest L_5_. Table 1 shows the position of the apex. Seven of 31 cases were complicated with L_4_ vertebral listhesis.

**TABLE 1:**
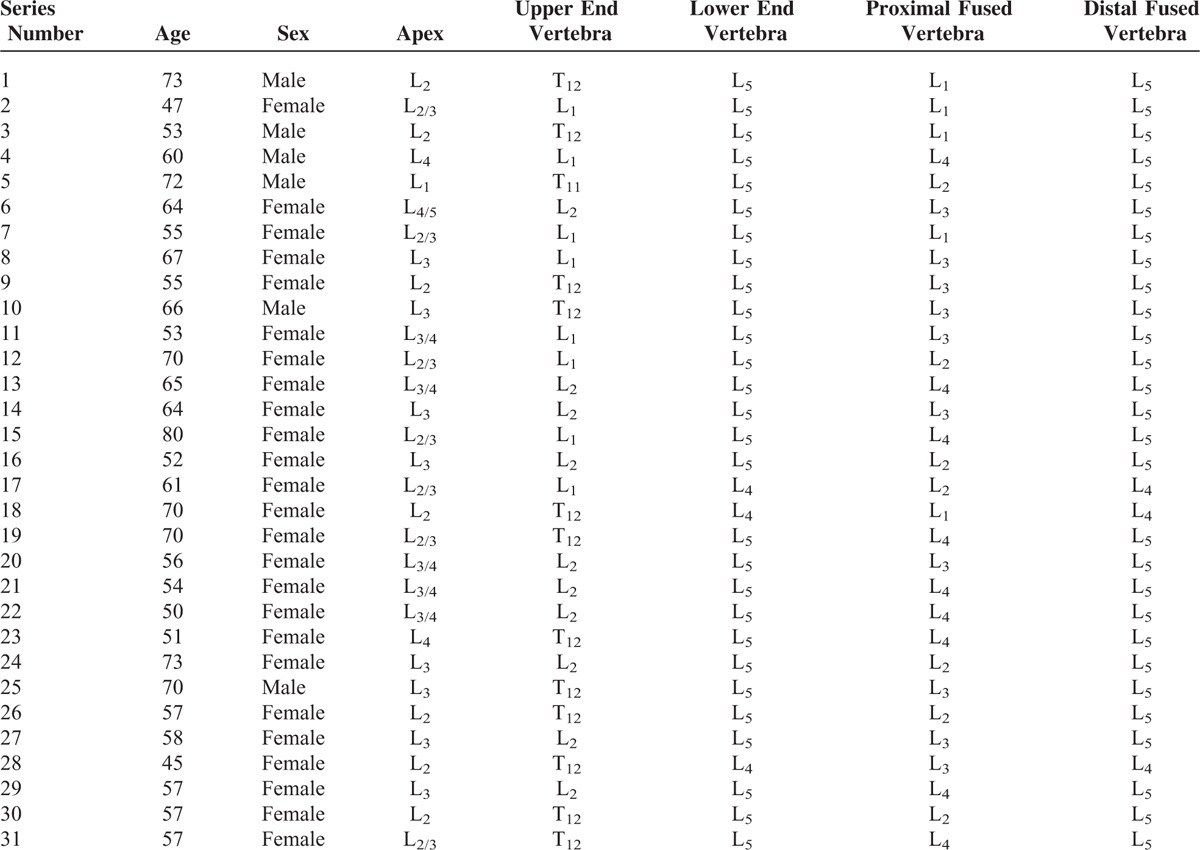
Basic Patient Information (31 Cases)

### Inclusion Criteria

Inclusion criteria includes the scoliotic Cobb angle >10°; the presence of prominent lumbar spine degeneration (intervertebral stenosis, articulate ossification and/or proliferation) complicated with lumbar spinal stenotic claudication; and the presence of radiating lower limb pain with intermittent claudication and painful positional low back pain.

### Exclusion Criteria

Exclusion criteria includes history of scoliosis (congenital or idiopathic scoliosis), history of lumbar spine surgery, congenital malformations of the spine, pelvis or lower limbs, spinal mass, and pathologic fracture of the spine. Besides, patients who were given sufficient conservative treatment before submitting them to operative intervention were excluded.

### Surgical Procedures

Thirty-one patients underwent posterior lumbar decompression and pedicle screw instrumentation with posterolateral bone graft fusion under general anesthesia in the prone position. According to the patient's osteoporosis situation, we have not carried out the forced correction and just made the appropriate corrections. Surgical indications were radiating pain and intermittent claudication after at least 6 months of ineffective conservative treatment. All patients underwent decompression at the stenotic levels, including 1 case at the L_1–4_ level, 4 cases at L_1–5_, 1 case at L_2–4_, 6 cases at L_2–5_, 1 case at L_3–4_, 9 cases at L_3–5_, and 9 cases at L_4–5_. The mean number of fused levels was 3.2 (range 2–5). The proximal fusion level was L_1_ in 5 cases, L_2_ in 7 cases, L_3_ in 10 cases, and L_4_ in 9 cases. The distal fusion level was L_4_ in 3 cases and L_5_ in 28 cases (Table 1).

**FIGURE 1:**
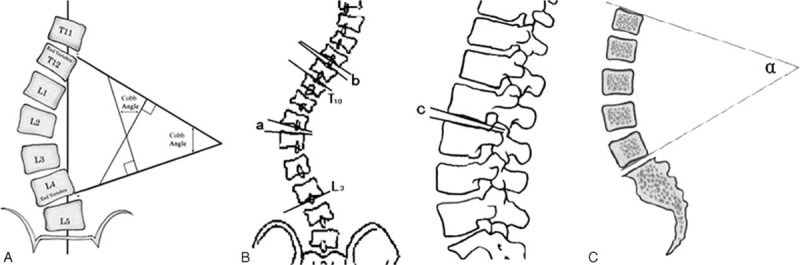
Radiographic measurements. (A) The coronal Cobb angle. (B) The intervertebral angle. On an anteroposterior x-ray, T_10_ is identified as the scoliotic UEV and L_3_ as the LEV; the scoliosis is toward the left, and the intervertebral angle (a) is unidirectional as the scoliotic angulations all open toward the left, providing a positive numerical value. The intervertebral angle (b) is in the opposite direction of the scoliosis curve angle, yielding a negative value. On the sagittal film, the intervertebral angle (c) is lordotic, again a positive value; if the angle is kyphotic, this value would be negative. (C) The lumbar lordosis angle. LEV = lower end vertebra; UEV = upper end vertebra.

Because patients mainly present with lumbar spinal stenosis, decompressive laminectomy is the preferred surgical treatment. Therefore, all of our patients underwent lumbar spinal decompression of the affected segment (using a posterior approach), pedicle screw internal fixation, and posterolateral bone graft fusion. The bone graft material is mainly derived from the autograft of the vertebral or the proliferation of articular. As mentioned previously, the decompressed and fused segment was localized to the levels affected by lumbar spinal stenosis. “Short segment fusion” indicates a fusion range that is limited to the entire length of the scoliotic segments and does not extend beyond the upper and lower end vertebra (EV).^14^ The intervertebral angle (IA) is defined as the angle formed by the upper terminal plate of the proximal fused vertebra and the lower terminal plate of the upper adjacent vertebra (Fig. 1B).^15^

Preoperative, postoperative, and follow-up examinations included a set of anteroposterior and lateral spinal x-rays, taken in the standing position. The scoliotic Cobb angle, the Cobb angle within the fusion levels, the anterior and sagittal intervertebral angles between the upper adjacent vertebra and the proximal fused vertebra, and the lumbar lordosis were measured (Fig. 1). Complications were recorded, including the development of bone graft fusion, pseudoarthrosis, or loosening or breakage of pedicle screws. Preoperatively, all patients underwent computed tomography (CT), computed tomography myelography (CTM), or magnetic resonance imaging (MRI) examinations to evaluate the degree of lumbar spinal stenosis and to aid in determining the fusion range.

The average follow-up period was 48.3 months (range 25–97 months). Follow-up clinical examinations and radiography were performed every 3 months for 3 years and every 6 months thereafter. The final follow-up was the patients’ last clinical examinations and radiography.

### Statistical Methods

Statistical analyses of preoperative, postoperative, and follow-up mean radiologic parametric comparisons were performed using SPSS 13.0 software by paired *t* test, with the level of statistical significance set at *P* < 0.05. We also performed parametric relativity analysis.

## RESULTS

Measurement and statistical analyses were performed for the collected preoperative, postoperative, and follow-up (3 months and final follow-up) radiological measurement data. The VAS (visual analog scale) and ODI (Oswestry Disability Index) for low back and/or leg pain were recorded preoperatively, 3 months postoperatively, and at the final follow-up.

The scoliotic Cobb angle decreased from 15.4° ± 5.3 preoperatively to 10.2° ± 5.3 immediately after surgery (Table 2), a difference that was statistically significant (*P* = 0.003). The average correction rate was 33.8%. The average correction loss was 4.4° at the final follow-up, and there was a statistically significant difference in terms of the immediate postoperative and final follow up results (*P* = 0.010). No significant differences for the scoliotic Cobb angle were found between the immediate postoperative and 3 months post-operative results (*P* = 0.205). Besides, no differences for the scoliotic Cobb angle were observed between the preoperative and final follow-up results (*P* = 0.647).

**TABLE 2:**
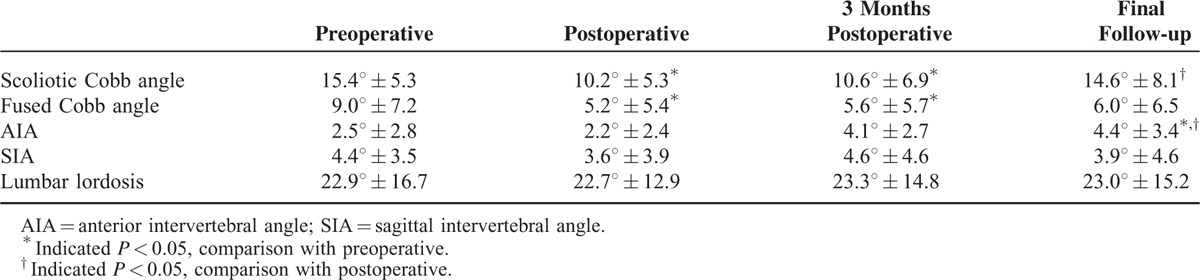
Pre/Postoperative Radiological Data (n = 31, x¯±s)

For the Cobb angle within the fusion levels, an obvious difference was found between the preoperative and immediate postoperative values (*P* = 0.018). There was also a significant difference between the preoperative and 3-month postoperative Cobb angles (*P* = 0.033). No significant differences were observed between the other time points for the Cobb angle within the fusion levels (*P* > 0.05) (Table 2).

For the AIA between the upper adjacent and proximal fused vertebra, there was a statistically significant difference between the preoperative, immediate postoperative, and final follow-up angles (*P* = 0.009 and *P* = 0.003, respectively) (Table 2). There were no significant differences in the AIA at other time points (*P* > 0.05).

There was no difference for the SIA between the upper adjacent and proximal fused vertebra for these comparisons (*P* > 0.05) (Table 2).

For the lumbar lordosis angle, there was no difference between the different time points (*P* > 0.05) (Table 2).

The assessments of lower back and leg pain according to the VAS and ODI demonstrated significant clinical differences in the preoperative, 3-month postoperative, and final follow-up scores (*P* = 0.000 and *P* = 0.000, respectively) (Table 3). No significant difference in the VAS or ODI scores was found between the 3-month postoperative and final follow-up assessments (*P* > 0.05).

**TABLE 3:**
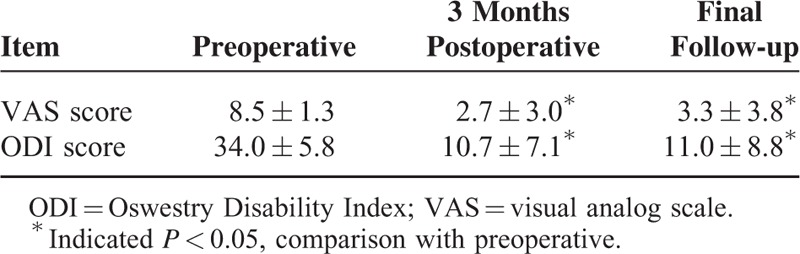
Pre/Postoperative VAS and ODI Scores (n = 31, x¯±ss)

Using bivariate Pearson analysis, we observed that postoperative decreases in the degree of the AIA and scoliosis correction were highly correlated (relativity values *r* = 0.745, *P* = 0.000). Additionally, the SIA was directly related to lumbar lordotic change (*r* = 0.614, *P* = 0.000). Visual analog scale and ODI improvements were directly related to changes in lumbar lordosis (*r* = −0.358, *P* = 0.048 and *r* = −0.326, *P* = 0.044, respectively). Using linear regression and Pearson analysis of the scoliotic Cobb angle, the AIA, the SIA, the lumbar lordosis angle, and patient age, we observed weak relationships between postoperative changes in the SIA and patient age (*r* = −0.339, *P* = 0.031). Other parameters were not related to the patient age.

### Complications

One patient, a 47-year-old woman who had undergone L_1–5_ internal fixation and fusion and L_4–5_ decompression, complained of low back pain and tenderness 71 months after the operation. X-rays indicated a broken pedicle screw at the L_5_ level on the left, with an obvious loss of scoliotic correction and lumbar lordosis. In elderly patients, pedicle screw breakage is mostly due to osteoporosis and vertebral compression, and loss of correction places increased loading pressure on the pedicle screws. After performing revision surgery, we extracted and exchanged the left L_5_ pedicle screw and left titanium rod.

Another patient, a 53-year-old man who had undergone L_1–5_ internal fixation and fusion, complained of severe lumbar discomfort and evident foreign object sensation. Bone fusion was observed 21 months following the initial operation; corrective surgery was performed to remove all internal fixation devices. This procedure completely alleviated the patient's symptoms.

None of the patients developed pseudoarthrosis, adjacent segment fractures, or other complications.

## DISCUSSION

Unlike idiopathic scoliosis in adolescents, which has obvious physical manifestations, surgical treatment is sought by adult patients with degenerative scoliosis for pain relief rather than cosmesis.^4,7^ There is still debate about the optimal surgical approach, and there is controversy over whether the proximal fusion level should extend beyond T10 and whether the L_5_/S_1_ intervertebral disc should be fused.^19–23^

Clinical studies have found that the incidence of pseudoarthrosis is higher at the thoracolumbar (TL) junction.^5,10–12^ Shufflebarger et al^5^ suggested that fusion at T_10_ or higher could prevent adjacent segment disease (ASD) because T_10_ has a true rib that can reinforce a susceptible TL junction. Cho^21^ also stated that one horizontal or neutral vertebra may help to determine the proximal fusion level in the coronal plane and that fusion to T_10_ is more stable than fusion to T_11_–L_1_. However, Shufflebarger et al^5^ stated that there is a lack of valid evidence supporting the efficacy of proximal fusion to T_10_ and above, contending that unnecessary fusion to T_9_ or T_10_ will increase the fusion level by 3 to 4 segments, resulting in increased bleeding and a longer operative time, which in turn increases the complication rate and the cost of surgery.

When selecting the distal fusion level, Hamill et al^20^ evaluated whether the intervertebral space between L_5_/S_1_ was relatively normal without disc degeneration. If lumbar lordosis and sagittal alignment are well maintained, extension of the distal fusion to L_5_ may be considered, preserving motion at L_5_/S_1_. Preservation of L_5_/S_1_ relies upon the quality of the intervertebral disc. Degenerative scoliosis often occurs in people at age ≥45 years, in whom concomitant L_5_/S_1_ intervertebral disc degeneration is prominent; therefore, fusion to L_5_ may result in high rates of revision surgery. As a result, various surgeons have suggested distal fusion to S_1_.^20,22,24^ S_1_ level fusion is indicated in patients with L_5_/S_1_ intervertebral disc degeneration; however, standards of care are still needed for such cases.

Liu et al^25^ stated that treatment should be individualized according to the patient's age, general and economic factors, the severity of the deformity, and the presence of other coexisting lumbar degenerative disorders. Long segment fusion, short segment fusion, and simple decompression without fusion are effective treatments for different forms of chronic degenerative lumbar scoliosis. However, in Liu et al's study, the Cobb angle and physiologic lordotic angle in patients who underwent multisegment (>3 segments) fusion improved to a greater extent than in patients who had simple decompression without fusion.^25^

Suk et al^14^ studied the effects of short and long fusion in patients with degenerative lumbar scoliosis, observing that long segment fusion provided better scoliosis correction and coronal balance, although it was less effective than short fusion in correcting lumbar lordosis and sagittal balance. The perioperative complications of long fusion included a high incidence of screw loosening and pseudoarthrosis, whereas patients undergoing short fusions had a higher risk of ASD.

Hwang et al^26^ reported that the scoliotic angle after short segment decompression and fusion does not seriously deteriorate in patients with degenerative lumbar scoliosis. They stated that a larger scoliotic angle and fusion to the apical vertebra are significant risk factors for the acceleration.^26^

In our study, among patients who underwent short fusion surgery for degenerative lumbar scoliosis, 71% of the proximal fused vertebra were at L_1–3_ (22/31 cases); therefore, the upper adjacent level was within the thoracolumbar junction. Biomechanically, this junction is relatively active and flexible compared to the more immobile thoracic spine. After short fusion, the load pressure was concentrated in the thoracolumbar junction. Additionally, short fusion provided little correction of the AIA, inducing uneven distribution of force and resulting in ASD, such as degeneration of the adjacent segment, exacerbation of scoliosis, compression fractures at the adjacent and proximal fused vertebra, failure of the proximal internal fixation, pseudoarthrosis, sagittal imbalance, and thoracolumbar kyphosis.^3,14,27^ In our study, 90% of the distal fusion levels were located at L_5_ (28/31 cases). From a biomechanical perspective, L_5_/S_1_ is in a leverage position and is a critical transitional point for lumbar motion between a stiff pelvic PA and the fused segment, which is also a stress point. If fusion to L_5_ does not routinely allow L_5_–S_1_ autocompensation, there will be increased stress on the L_5_/S_1_ intervertebral disc and articular facets, accelerating degeneration, and increasing the incidence of related symptoms.^18,23,26^ However, we observed that 22 patients with proximal fused vertebra at L_1–3_ did not have the ASDs mentioned above, and the 28 patients who underwent distal fusion to the L_5_ vertebrae did not present with L_5_/S_1_ intervertebral disc degeneration that required revision surgery. This differs from previous reports of short segment fusion^22,23^ and could be due to the shorter follow-up time; further research is needed to address this issue.

Hwang et al^26^ studied short segment decompression and reported that fusion could correct scoliotic curvature to a small extent. The authors stated that deformity corrections and long segment fusions are appropriate for patients with a large Cobb angle or sagittal imbalance.^26^

**FIGURE 2:**
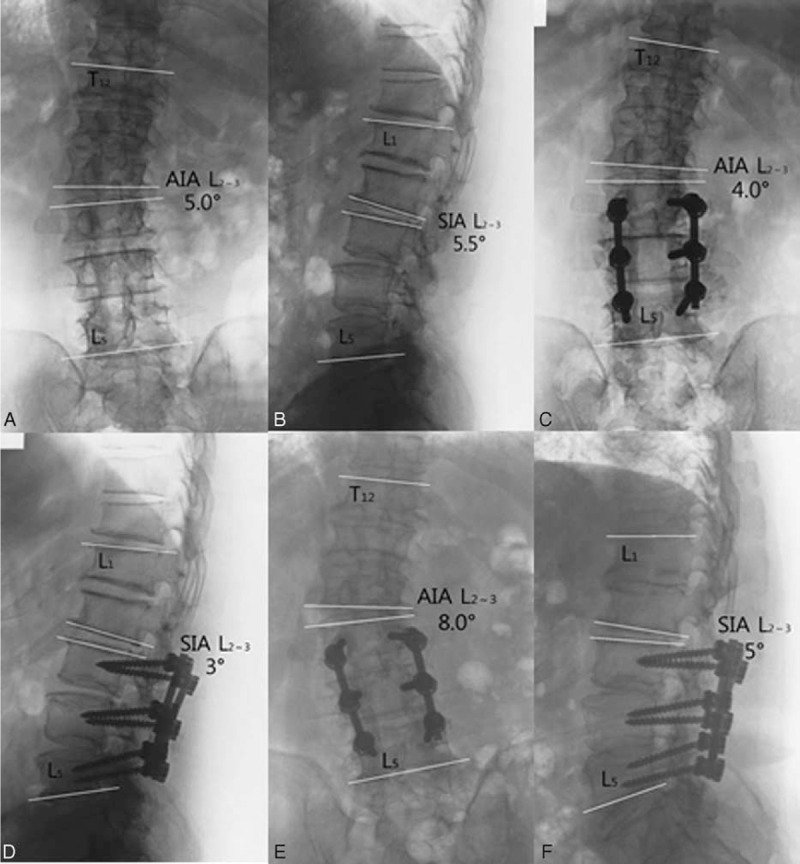
A 70-year-old man who had low back pain for >20 years with intermittent claudication for 5 years was diagnosed with degenerative lumbar scoliosis and lumbar spinal stenosis. Preoperative findings included a scoliotic Cobb angle T_12_–L_5_ of 13.5°, an AIA L_2–3_ of 5.0°; at fusion level L_3–5_, the Cobb angle was 3.0°, the SIA L_2–3_ 5.5°, and the lumbar lordosis L_1–5_ 11° (A, B). The patient underwent L_3–5_ internal fixation and decompression; postoperatively, the scoliotic Cobb angle was 15.5°, the AIA L_2–3_ 4.0°, the fusion level L_3–5_, Cobb angle 5.0°, the SIA L_2–3_ 3°, the lumbar lordosis 14° (C, D); and 31 months postoperatively, the scoliotic Cobb angle was 18°, the AIA L_2–3_ 8.0°, the fusion level L_3–5_, Cobb angle 5.5°, the SIA L_2–3_ 5°, and the lumbar lordosis 20° (E, F). AIA = anterior intervertebral angle, SIA = sagittal intervertebral angle.

According to the results of our study, short fusion allowed for a 33.8% scoliosis correction rate in degenerative lumbar scoliosis, with a relatively limited correction that could be due to the reduced correction force of the short fusion. However, we must not exclude the possibility of more rigid degenerative lumbar scoliosis. Upon follow-up, there was an observable loss of correction, which increased over time. At the time of the final follow-up, the average correction loss was 4.4°, with simultaneous worsening of the AIA, which exhibited an average increase of 2.2° at the final follow-up. The change in the AIA was directly related to the loss of scoliotic correction, indicating coronal deterioration and loss primarily in segments that were not included in the fusion levels (Fig. 2). The following factors may help to explain these observations. First, the thoracolumbar junction was originally under a heavy load and force; after short fusion with insufficient correction, global spinal stability and balance were not achieved, and the AIA could not be corrected. Additionally, postoperatively, an asymmetric stress force is introduced to the adjacent intervertebral disc and facet joint, resulting in degeneration of the adjacent segment and deterioration of the AIA.^3,19,28,29^ This is a domino effect caused by the increased AIA, resulting in worsening scoliosis. However, our patients did not complain of discomfort nor did they present with complications related to the AIA increase. Furthermore, no patients required revision surgery for the AIA increase, which could be related to the shorter follow-up times.

The results of our study indicated that short segment fusion did not affect the SIA, which may be due to degenerative scoliosis that mainly presents with coronal instead of sagittal misalignment. Lumbar lordosis was also unaffected by short segment internal fixation with insufficient correction of lumbar lordosis and limited sagittal balance impact.^3^ Most patients who developed decreases in the SIA were female; they were also more likely to be older and postmenopausal with various degrees of osteoporosis, resulting in a higher incidence of osteoporotic wedge vertebrae, collapsed vertebrae, and stress fractures.^3,10,12,30^ However, the statistical power of our analysis is limited given the small sample size of our study. After careful analysis, the progression of the SIA was associated with the change in lumbar lordosis (*r* = 0.614, *P* = 0.000) as well as patient age (*r* = −0.339, *P* = 0.031); predisposing osteoporosis, vertebral collapse, and disc degeneration all directly affect the SIA and kyphotic changes.^10,12^

Our analysis showed that the lumbar pain VAS and ODI scores were related to lumbar lordosis, which is consistent with prior publications.^8,31^ However, the VAS and ODI scores were not related to scoliosis correction, the loss of correction, the AIA or SIA. This indicated that short fusion resulted in limited correction, the loss of correction, and increasing exacerbation of the AIA and SIA; however, short fusion did not appear to cause obvious clinical manifestations or symptoms. None of the patients in our study required revision surgery for the loss of correction or exacerbation of the IA.

Theoretically, in patients who undergo short fusion, the incidence and degree of adjacent segment degeneration may progressively increase with sufficient follow-up time. However, most cases of degenerative scoliosis are in elderly patients with limited life expectancies, such as those included in our study (mean age 60.7 years, with the oldest patient 80 years old). Daily exercise typically decreases with increasing age; therefore, it is unlikely that ASD resulting in serious clinical complications and requiring revision surgery would occur during the remainder of such patients’ life expectancies. However, validation of these findings requires longer follow-up in future studies.

There were certain defects in this study, of course. The heterogeneity between individual studies was substantial because the indications for surgery, surgical procedures, and outcome measures varied among the studies. The circumstances of long-term complication were not clear due to the insufficient follow-up duration. Besides, because of the limited condition and time, the number of patients is not large enough, which may affect the accuracy of result. Therefore, these aspects will be the key targets for observation in our future researches.
